# Muscle Activation of Vastus Medialis Oblique and Vastus Lateralis in Sling-Based Exercises in Patients with Patellofemoral Pain Syndrome: A Cross-Over Study

**DOI:** 10.1155/2015/740315

**Published:** 2015-10-04

**Authors:** Wen-Dien Chang, Wei-Syuan Huang, Ping-Tung Lai

**Affiliations:** ^1^Department of Sports Medicine, China Medical University, No. 91, Hsueh-Shih Road, Taichung 404402, Taiwan; ^2^Department of Physical Therapy and Rehabilitation, Rehabilitation Assistive Device Center, Da-Chien General Hospital, No. 6, Shin Guang Street, Miaoli City 36049, Taiwan

## Abstract

*Objectives*. To examine what changes are caused in the activity of the vastus medialis oblique (VMO) and vastus lateralis (VL) at the time of sling-based exercises in patients with patellofemoral pain syndrome (PFPS) and compare the muscular activations in patients with PFPS among the sling-based exercises. *Methods*. This was a cross-over study. Sling-based open and closed kinetic knee extension and hip adduction exercises were designed for PFPS, and electromyography was applied to record maximal voluntary contraction during the exercises. The VMO and VL activations and VMO : VL ratios for the three exercises were analyzed and compared. *Results*. Thirty male (age = 21.19 ± 0.68 y) and 30 female (age = 21.12 ± 0.74 y) patients with PFPS were recruited. VMO activations during the sling-based open and closed kinetic knee extension exercises were significantly higher (*P* = 0.04 and *P* = 0.001) than those during hip adduction exercises and VMO : VL ratio for the sling-based closed kinetic knee extension and hip adduction exercises approximated to 1. *Conclusions*. The sling-based closed kinetic knee extension exercise produced the highest VMO activation. It also had an appropriate VMO : VL ratio similar to sling-based hip adduction exercise and had beneficial effects on PFPS.

## 1. Introduction

Patellofemoral pain syndrome (PFPS) is a musculoskeletal disorder that often occurs in the lower extremities of athletes and has a high prevalence in women aged 18–35 years [1]. Although the causes of PFPS are not clearly understood, this syndrome is related to abnormal patellar arrangement and biomechanics, for example, increased *Q* angle, patellar maltracking, excessive foot pronation, and excessive external torsion [1]. Because of muscle imbalance between the vastus medialis oblique (VMO) and vastus lateralis (VL), the VMO cannot antagonize the VL, resulting in patellar maltracking. Empirical evidences represented that an imbalance of VMO and VL muscle activities leads to excessive lateral tracking of the patella and rubbing of the lateral femoral condyle, which causes articular surface erosion and degeneration and induces pain [2].

Different strengthening techniques have been suggested for VMO training, which can be categorized into open or closed kinetic chain exercises. Previous study demonstrated that knee extension exercises with knee flexion angles between 0° and 60° were recommended for VMO activation [3]. During a knee extension exercise, the patella is pulled by the quadriceps muscle and the cranial slides within the femoral trochlear groove, and the exercise is an efficacious treatment for an imbalance of VMO and VL muscles, which is thought to be present in patients with PFPS, to stabilize patellar tracking [2]. The electromyography (EMG) study represented that the ideal VMO : VL ratio for healthy individuals during knee extension is 1 : 1 [4]. Previous study has indicated that the value of VMO : VL ratio in patients with PFPS was 0.54 : 1 [5]; this is possibly because imbalance of VMO and VL muscle activities causes insufficient medial patellar strength, which induces patellar maltracking in PFPS [4]. An evidence-based approach indicated that VMO training, using open or closed kinetic chain exercise, effectively prevents and alleviates PFPS [6]. Hip adduction exercise is another specific therapeutic exercise for PFPS patients. It can facilitate increasing VMO muscle activity because the origin of VMO muscle connects to the insertion of the hip adductor muscles [7]. The hip adductor muscle training is often added to rehabilitation program of PFPS, because it can facilitate VMO contraction, which is preferentially recruited in hip adduction exercises [8]. This is a finding supported by Earl et al. [9], and they combined closed kinetic squats with isometric hip adduction exercises and found that the integrated movement more efficiently recruited quadriceps activity than did closed kinetic squat exercises alone. Hip adduction exercise is also suggested for patients with PFPS. The presence of effective therapeutic exercises of knee extension and hip adduction creates a dilemma for treating the patients with PFPS. It is unclear what a suitable and safe movement of the exercises can improve VMOmuscle strength. VMO activity and VMO : VL ratio of EMG may be effective indicators to assess the training effect.

Sling exercise training is a multifunctional training program involving various exercises for athletes. During sling exercise training, exercise instructors can adjust suspensory points, positions of a suspended body, and sling height. Sling equipment, such as elastic ropes, cushions, and weights, can be used for achieving physical fitness, such as increasing muscle strength and endurance, improving balance and proprioception, and stabilizing core muscles [10]. Sling exercise training is widely used in rehabilitation clinics and fitness training centers for rehabilitating patients and injured athletes [11]. For clinicians treating patients with PFPS, sling exercise training may be used for designing closed and open kinetic chain exercises for 0°–60° knee extension and hip adduction movement. However, it is unknown whether sling-based knee extension or hip adduction exercise will better influence quadriceps muscle contraction. Although sling-based exercise has the potential to be a conservative treatment for PFPS, the evidence of VMO activation has yet to be validated and, therefore, provide little clinical use. The aims of this study were to investigate the change of three sling-based exercises in activity of the VMO and VL muscles and compare the muscular activation in patients with PFPS.

## 2. Methods

### 2.1. Subject Selection

The current study was a cross-over design. Patients were recruited in the rehabilitation center of Dachien Teaching Hospital from January 2012 to December 2014. They were clinically diagnosed with PFPS by a physician via presentations of a positive patellar compression test or tenderness around the patella with palpation and occurrences of anterior knee pain during ascending and descending stairs, or prolonged sitting. Inclusion criteria were the presence of anterior knee pain lasting longer than 6 months, and two of the following symptoms without traumatic injury, that is, knee pain when climbing stairs or during squatting, kneeling, lengthy sitting, hopping, and jumping. Patients were excluded if they had any recent surgery of knee or lower extremity, and their body mass index (BMI) was higher than 30 [12]. On that test day, they were instructed to avoid taking all anti-inflammatory drugs, and other treatments, such as physical therapy and other conservative treatments, were also disallowed before the experiment. The patients were recruited and the affected and dominant legs were tested [12]. The dominant leg is the leg used to kick a ball and the first leg used to step onto stairs. Visual analog scale is used to assess presently perceived pain in activity of daily life before starting the study. The medical ethics committee of Dachien Hospital approved this study. Participants were informed of the purpose and risks of the study, and they signed consent forms. Sample size was performed using the G^*∗*^Power software (G^*∗*^Power 3.1.9.2, Heinrich-Heine-Universität Düsseldorf, Germany) and estimated using the data in the pilot study from 7 patients (3 male and 4 female patients) with PFPS. The means and standard deviations of the assessed variables among the sling-based exercises were calculated. The calculations were based on detecting a mean difference in the assessed variables of EMG between the exercises, with standard deviation of difference, which was between 0.09 and 0.16, at the pilot result. In the current study, estimated sample size with a power = 0.80 and *α* = 0.05 was between 12 and 36 per sling-based exercise [13].

### 2.2. Assessment Tool

**Figure 1:**
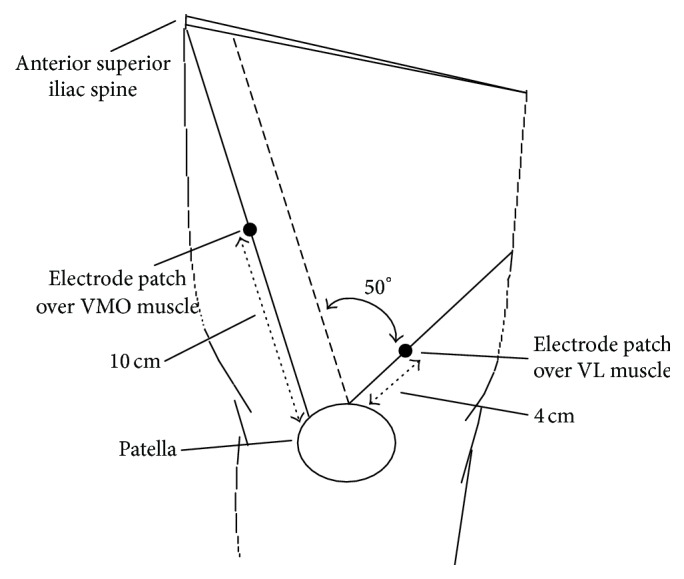
Electrode placement.

Surface EMG (MyoTraceTM 400, Noraxon Inc., USA) and electrode patches (Medi-Trace 200, Kendall, USA) with 20 mm interelectrode separation were used. For the VL, an electrode patch was placed on the line between the outer side of the patella and the anterior superior iliac spine and was located 10 cm from the patella. For the VMO, an electrode was placed on the line forming a 50° angle with the parallel line between the outer side of the patella and the anterior superior iliac spine and was located 4 cm from the patella [14], as shown in Figure 1. An electrogoniometer (NorAngle Electrogoniometer System, Noraxon Inc., USA) recorded the range of knee extension using the lateral femoral condyle as the axis. EMG signals were collected by an examiner (P.-T. L.), and data were analyzed and assessed by another examiner (W.-D. C.). The fixed arm was aligned with the midline of the lateral thigh, and the mobile arm was aligned with the midline of the lateral crural region and firmly secured to the skin surface. Surface EMG recordings when the exercises had well excellent test-retest reliability and high intraclass correlation coefficients were 0.89 for the VMO and 0.95 for the VL [15].

### 2.3. Sling-Based Exercises

**Figure 2:**
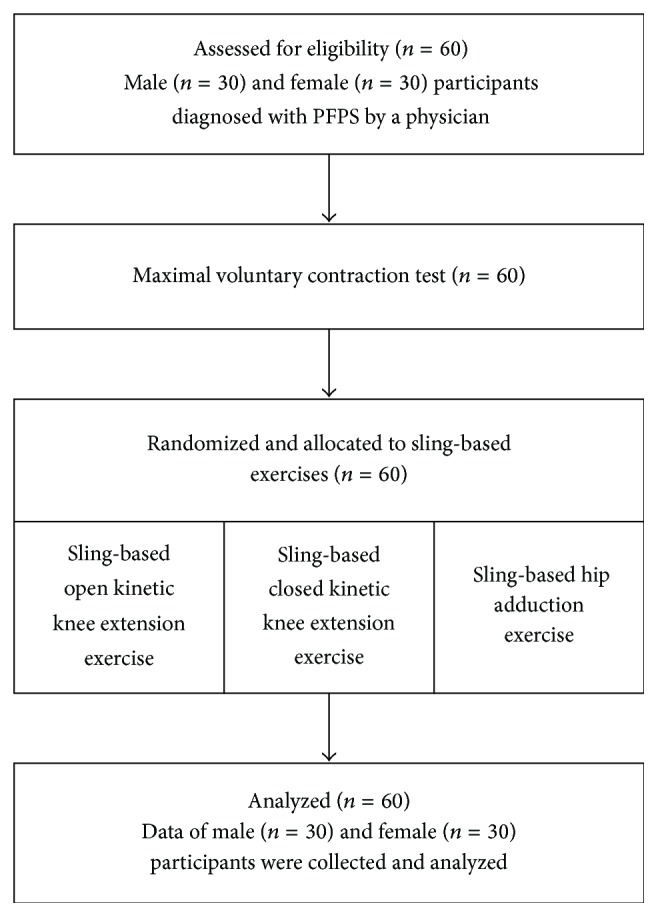
Participant flow diagram.

The participants chose the sequence to perform three sling-based exercises (sling-based open kinetic knee extension, sling-based closed kinetic knee extension, and sling-based hip adduction exercises) in random order using a table of random numbers. The flowchart diagram of the participants' progress was shown in Figure 2. The exercises were guided by the same therapist (P.-T. L.) and described as follows.

**Figure 3:**
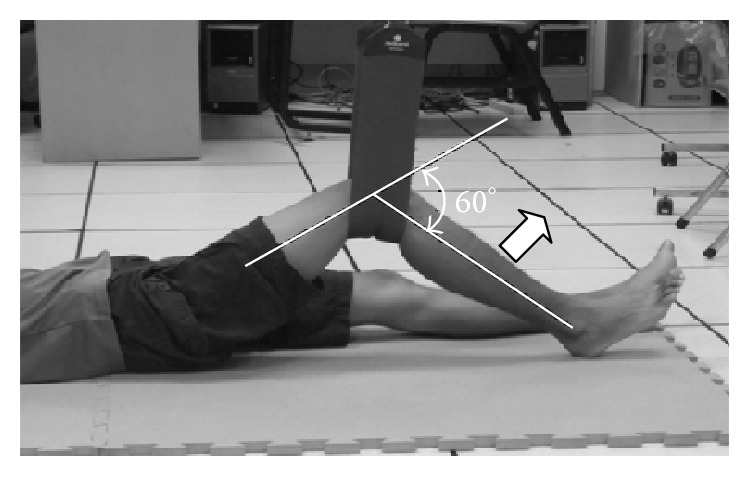
Sling-based open kinetic knee extension exercise.

*(1) Sling-Based Open Kinetic Knee Extension Exercise.* Participants lay on the floor with both hands at the sides of the body. A sling was placed on the popliteal fossa. Participants were requested to straighten their lower legs from a knee flexion angle of 60°, hold for 5 s, and relax the legs to the starting position (Figure 3).

**Figure 4:**
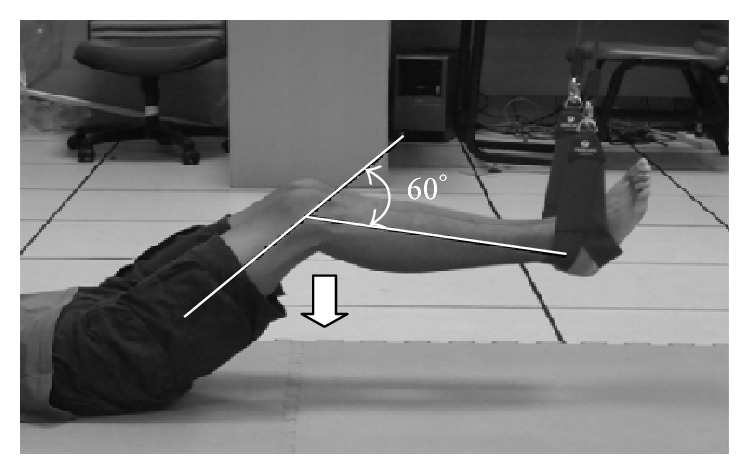
Sling-based closed kinetic knee extension exercise.

*(2) Sling-Based Closed Kinetic Knee Extension Exercise.* Participants lay on the floor with both hands at the sides of the body. Both ankles were placed in slings. Participants were requested to raise their pelvis, press the knees down, straighten the legs from a knee flexion angle of 60°, hold the position for 5 s, and relax the legs to the starting position (Figure 4).

**Figure 5:**
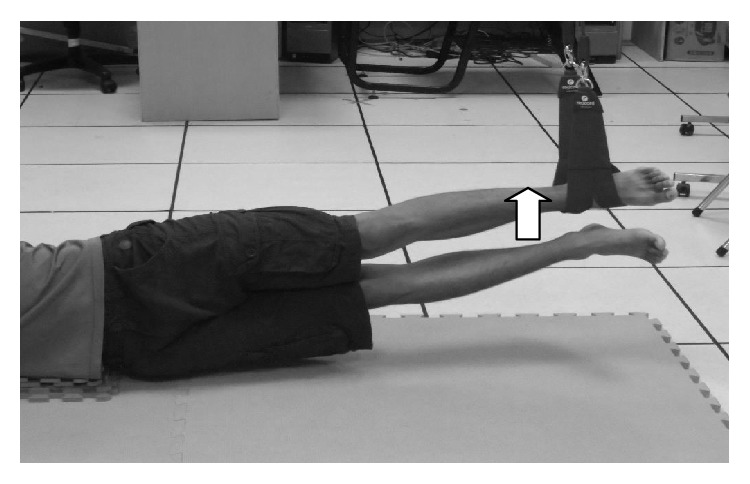
Sling-based hip adduction exercise.

*(3) Sling-Based Hip Adduction Exercise.* Participants lay on the floor and straightened their legs with their hands placed at the sides of the body. The sling was placed on the unaffected ankle. Participants were requested to lift the affected leg, hold for 5 s, move toward the unaffected leg, execute a hip adduction movement, and relax to the starting position (Figure 5).

### 2.4. EMG Signal Processing

Participants were briefed on the training method in the laboratory of rehabilitation center, and VMO and VL electrode placements were marked on their affected and dominant legs. After the marked areas were wiped clean by using alcohol swabs to reduce skin impedance interference with EMG signals, the electrode patches were placed and connected to the EMG. Participants drew lots to determine the sequence of the three sling-based exercises.

To standardize the EMG signals induced by the three exercises, a maximal voluntary contraction (MVC) test during the knee extension resistance exercise was performed before the sling-based exercises. Participants were required to sit on a high platform without touching the floor, and their popliteal fossae were placed against the edge of the platform at a knee flexion angle of 90°. They were requested to lift affected legs to a knee flexion angle of 60° and to exert maximum effort in straightening the legs. The examiner's hand was placed on the test leg's ankle to antagonize the ankle and cause maximal isometric contraction of knee extension. The test was repeated 3 times with a 10 s break between repetitions to avoid muscle fatigue. Three values, MVCs of the VMO and VL and the value of VMO : VL ratio, were analyzed; the original EMG signals were processed and analyzed for determining the MVCs of VMO and VL. EMG signals were processed at a sampling rate of 2000 Hz, and a 10–500 Hz bandpass filter was used to remove the external noise. The original EMG signals underwent full-wave rectification, and the obtained absolute values were treated with an antialiasing technique (20 ms root mean square). Finally, amplitude normalization at 300 ms yielded MVC values for maximal isometric contraction of knee extension. A percentage of the MVC recorded during the exercises was represented as the muscle activity. For standardization, MVC values were converted to the percentage of the MVC as follows: MVC = (MVC for sling-based exercise/average MVC of maximal isometric test for knee extension) × 100.

After the MVC test, participants took a 10 min break and were subsequently trained in the 3 sling-based exercises. Each movement was repeated 3 times with a 10 s break between repetitions. The participant took 10 min breaks between each training session.

### 2.5. Statistical Analysis

The data obtained in this study were analyzed using SPSS Version 17 (SPSS Inc., Chicago, IL, USA). Descriptive statistics (i.e., mean and standard deviation) of the participants' demographic data (age, height, weight, and BMI) and assessed variables were calculated, and Kolmogorov-Smirnov test was used to determine normal distribution. Independent *t*-test was used for comparison between male and female participants for the demographic data. Repeated-measures analysis of variance and post hoc tests were conducted to determine whether significant differences in the MVCs of the VMO and VL and VMO : VL ratio exist among the three exercises (i.e., sling-based open and closed kinetic knee extension and hip adduction exercises). A two-tailed test with *α* = 0.05 was performed. The consistency of activation of VMO and VL for the maximum contraction among the 3 repetitions of each exercise for each individual was analyzed using the intraclass correlation coefficient (ICC).

## 3. Results

Thirty male (average age = 21.19 ± 0.68 y, average height = 168.57 ± 4.7 cm, average weight = 63.34 ± 6.4 kg, and average BMI = 21.16 ± 2.04) and 30 female (average age = 21.12 ± 0.74 y, average height = 161.73 ± 4.5 cm, average weight = 52.64 ± 5.3 kg, and average BMI = 20.26 ± 2.12) participants were recruited. They were recruited in the rehabilitation center, and the average onset of PFPS that had occurred was 7.94 ± 2.86 months. All of them had completed the experimental procedure without dropping out the study. The height, weight, and BMI of male participants were significantly higher than those of female participants except for the age (*P* > 0.05). The right leg was the affected and dominant leg of all the participants. Visual analog scale was 1.5 ± 1.8, and that did not affect the exercises performance. The ICC of the VMO and VL activation for 3 repetitions' maximum contraction was 0.92 ± 0.13, and no significant differences existed in the three exercises among the 3 repetitions. The repeated-measures analysis of variance showed that the type of the sling-based exercise had significant relation with the MVCs of the VMO and VL and VMO : VL ratio (*P* < 0.05).

### 3.1. VMO and VL Electrical Activity

No significant differences were observed in the VMO and VL activation between males and females for the three exercises (*P* > 0.05) except the VL activation for the hip adduction exercise (*P* = 0.04). The VMO activation during the sling-based open and closed kinetic knee extension exercises (*P* = 0.04 and *P* = 0.001) was significantly higher than that during the hip adduction exercise (Table 1). A significant difference in the VMO activation was observed between the sling-based open and closed kinetic knee extension exercises (*P* = 0.007). The VL activation during the sling-based open and closed kinetic knee extension exercises (*P* = 0.001 and *P* = 0.001) was significantly higher than that during the hip adduction exercises (Table 1). No significant differences in VL activation were observed between the sling-based open and closed kinetic knee extension exercises (*P* = 0.08).

### 3.2. VMO : VL Ratio

**Figure 6:**
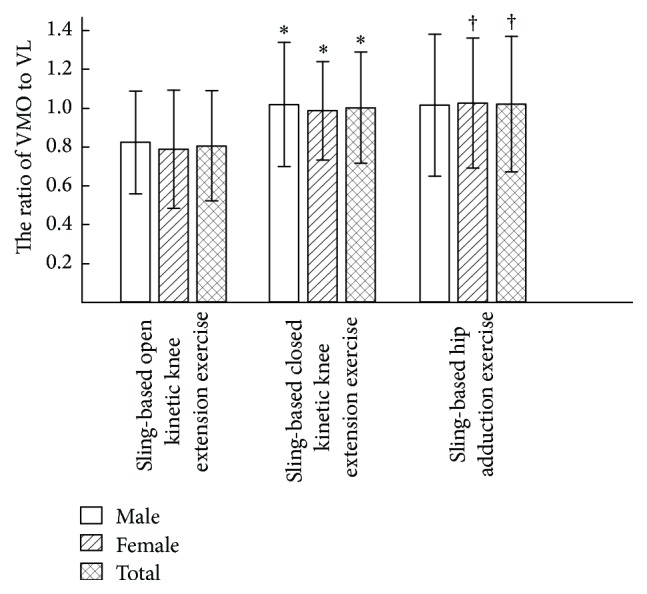
VMO : VL ratio for the three exercises. ^*∗*^Significantly greater muscle activation (*P* < 0.05) compared with the sling-based open and closed kinetic knee extension exercises. ^†^Significantly greater muscle activation (*P* < 0.05) compared with the sling-based open kinetic knee extension and hip adduction exercises.

No significant differences were observed in the values of VMO : VL ratio between males and females for the three exercises (*P* > 0.05). The VMO : VL ratios of the male participants for the sling-based open and closed kinetic knee extension and hip adduction exercises were 0.81 ± 0.34, 1.01 ± 0.32, and 1.01 ± 0.36, respectively, and the corresponding ratios for the female participants were 0.78 ± 0.30, 0.98 ± 0.25, and 1.02 ± 0.61, respectively. For all participants, the corresponding ratios were 0.80 ± 0.31, 1.00 ± 0.28, and 1.02 ± 0.35, respectively. Compared with the sling-based open kinetic knee extension exercise, the VMO : VL ratios for the sling-based closed kinetic knee extension and hip adduction exercises were relatively higher (*P* = 0.001 and *P* = 0.001). No significant differences were observed in the VMO : VL ratios of the sling-based closed kinetic knee extension and hip adduction exercises (*P* = 0.76), as shown in Figure 6.

## 4. Discussion

The device of sling exercise training is convenient and novel and currently is used in numerous fitness training centers [16]. No research, as yet, has investigated the activation of the medial and lateral muscles during sling exercise training for managing PFPS. The current study addressed this topic, and our results of EMG indicated that the sling-based open and closed kinetic knee extension exercises are substantially useful in VMO activation and that the sling-based open and closed kinetic knee extension exercises are substantially useful in VL activation. These results are consistent with those of previous study [17]. The results of our study found that sling-based open kinetic knee extension exercise had lesser VMO : VL ratio and had little beneficial effect on PFPS, because it may cause excessive lateral patellar tracking [18]. A recent study indicated that the reason is that open kinetic knee extension exercise is not a functional intervention, and not similar to closed kinetic knee extension, which can utilize muscle cocontraction and proprioceptive reaction of multiple joint [19]. On the contrary, the VMO : VL ratios for the sling-based closed kinetic knee extension and hip adduction exercises mostly approximate to 1 and differ significantly from those for the sling-based open kinetic knee extension exercise. The sling-based closed kinetic knee extension and hip adduction exercises are potentially important exercises and can be useful in training quadriceps strength of the PFPS rehabilitation. A hip adduction exercise adding to the closed kinetic knee extension exercise is also suggested in the rehabilitation program and is beneficial to patients with PFPS, because previous studies results showed that the exercises offer ideal value of VMO : VL ratio and increase quadriceps muscle activity [20, 21].

Open and closed kinetic chain exercises of knee extension are therapeutic exercises for PFPS. Regarding open kinetic chain exercise, the distal components of limbs are not fixed and joints move freely without support from other joints. Closed kinetic chain exercise is a movement where the distal components of limbs are fixed and joints require support from other joints [3]. Closed kinetic knee extension exercises cause quadriceps muscle contraction, which enhances knee joint stability. Open kinetic knee extension exercises are frequently used in the early stages of knee injury rehabilitation. Compared with closed kinetic knee extension exercises, open kinetic knee extension exercises are safer quadriceps-strengthening interventions for patients with anterior cruciate ligament injuries [22, 23]. Accounting for both protection of the anterior cruciate ligament and obtainment of muscle strength, open kinetic knee extension exercises can increase more activation of quadriceps muscle without causing an anterior shearing force of tibiofemoral joint. Closed kinetic chain exercises induce patellofemoral stresses in obese individuals and are unsuitable for patients with a high risk of falls [24]. Muscle strength training combining open or closed kinetic chain exercises with sling exercise can increase more creativity for the patients and decrease weight-bearing on knee joint. Sling exercise training provides active movement, where muscle contraction is aided by the sling exercise equipment [16]. Altering the sling points and heights of the dangling rope in sling-based training helps patients train specific muscle groups and provides an unstable surface where muscle groups receive antigravity training. In sling-based training, a sling supports the weight of various body parts; therefore, compared with traditional training, it is an easier program for patients to improve body trunk muscle strength [25]. Sling-based training was often practiced to train core muscle groups, such as the chest, waist, and abdomen, and to improve muscle strength and stability of the body trunk [25, 26]. To the best of our knowledge, this is the first study to investigate the application of sling-based training to PFPS-affected lower extremities. Dannelly et al. [3] designed closed kinetic exercises on the basis of sling exercise training and determined that sling-based closed kinetic exercise substantially affected the muscle strength of the lower extremities. Some studies have indicated that sling exercise training routines used in open and closed kinetic chain exercises were effective in training muscle contraction through gradual resistance [26, 27]. Sling exercise training entails sensorimotor exercises and can be employed to increase neuromuscular activation through dynamic contraction exercises [28], which possibly increase the VMO and VL activation during sling-based open and closed kinetic exercises.

Irish et al. [18] determined that traditional closed kinetic exercises and sling-based open and closed kinetic knee extension exercises effectively increased the VMO activation. Regarding VL activation, traditional open and closed kinetic exercises did not differ significantly. Ott et al. found that patients with PFPS had decreased VMO and VL activation during anterior knee pain increasing [29]. For sling-based movements, both open and closed kinetic knee extension exercises effectively increased the VL activation, and this is possibly because sling-based knee extension exercises require lifting the pelvis, positioning it in line with the body, and maintaining the posture on unstable surfaces [30]; this causes muscle contraction in other parts of the body while maintaining body balance, which affects VL and VMO control. Hence, open and closed kinetic knee extension exercises should be used to effectively train the VMO and VL. Dannelly et al. indicated sling-based exercise is the convenient and effective strength training either in open or in closed kinetic exercise programs [3]. Our results also represented that sling-based closed kinetic knee extension exercise created a significantly greater VMO activation than the other sling-based exercises. The sling-based closed kinetic knee extension exercise produced significantly lesser VMO activation than sling-based open kinetic knee extension exercise, resulting in the less VMO : VL ratio. It can be anticipated that the sling-based closed kinetic knee extension exercise can be useful to increase muscle strength of VMO for patients with PFPS. Previous studies also confirmed that closed kinetic knee extension exercise elicits sport performance and strength better than open kinetic knee extension exercise [30, 31]. The sling-based closed kinetic knee extension exercise could be applicable and effective in PFPS rehabilitation.

During knee bending, the patella slides against the femoral groove and the VMO and VL concurrently help maintain patellar stability at the center of the femoral groove [32]. When the unbalanced strengths of VMO and VL muscle decreased the patellar dynamic stability, the patella is affected by the VL and moves outward of femoral groove [21]. Surface EMG receives the myoelectric signals generated by muscle contraction, which are detected by the electrode patches attached to the muscle belly. The EMG signal amplitude represents the level of muscle activation, and higher amplitude indicates the recruitment of a higher number of motor units [33]. MVC values can be used to quantify muscle activation and determine the imbalance of VMO and VL muscle in patients with PFPS [17] and can also be used to assess the effectiveness of exercise training [34]. The VMO : VL ratio represents the balance of VMO and VL activation, which provides stable force to patella. Powers determined that, for patients with severe PFPS, VMO : VL ratio was typically less than 0.54 [5]. Souza and Gross [4] stated that the ideal VMO : VL ratio is 1, which indicates that the patella does not slide off the femoral groove, and therefore PFPS does not occur. Thus, ideal VMO : VL ratio derived for various knee exercises is crucial in rehabilitation program for patients with PFPS. They also indicated that, in traditional closed kinetic chain exercises, VMO : VL ratio mostly approximates to 1. Tang et al. [19] compared the VMO : VL ratio of concentric and eccentric contractions in traditional open and closed kinetic chain exercises at various angles. They determined that the VMO : VL ratio observed during concentric contraction at a knee flexion angle of 60° in closed kinetic knee extension exercise was closer to the ideal value than that observed in open kinetic knee extension exercise. Sling-based hip adduction exercises were expected to increase the VMO activation; however, the results indicate that the VMO activity level is similar to the VL activity level and that the VMO : VL ratio approximates to 1. The study presented some limitations which should be comparing the training effects for sling-based exercises and the traditional kinetic chain exercises and lack of blinding in the present trial. VMO and VL activation and VMO : VL ratio at various angles of knee flexion needed to be analyzed among the sling-based exercises. These results maybe provide more inferences to prove the effect of sling-based exercise on PFPS in the clinical practice.

Previous study has shown that isometric contractions of either weight-bearing knee flexion or seated hip adduction can promote VMO contraction [32]. Compared to sling-based open and closed kinetic knee extension exercises, the results of this study showed that the MVC of the VMO does not exceed that of the VL and that VMO : VL ratio is close to 1, which does not affirm that sling-based hip adduction exercises can increase VMO activation. Because VMO originates from the insertion of the hip adductor muscle, the VMO can be stabilized by contracting the adductor muscles. Hip adduction exercises can stretch the VMO and improve its contraction strength and muscle tension [8]. Earl et al. [9] observed that squats in addition to hip adduction were more effective in recruiting quadriceps than only squats. Irish et al. [18] reported that double-leg squats combined with isometric contraction of hip adduction induced higher VMO recruitment than the lunge exercise. Felício et al. [35] proposed that knee exercises, including squats and knee extension, combined with hip adduction enhance VMO activity. The reason for additional hip adduction increasing VMO contraction strengths is that it changes in the muscular length and tension relationship [20, 35]. Hence, additional hip adduction has been recommended for improving the balance of VMO and VL. Coqueiro et al. [24] determined that patients with PFPS had more VL activity when subjected to closed kinetic squat adding hip adduction isometric contraction than when subjected to squats alone. Squats alone lead to more activity in the VL than in the VMO, subsequently leading to imbalance in the quadriceps. However, the present study determined that sling-based closed kinetic knee extension exercises lead to more activity in the VMO than in the VL. Sling exercise training destabilizes the distal lower extremity during knee extension exercises, reduces the weight-bearing loading, and reduces VL recruitment. This mechanism helps patients with PFPS effectively train VMO contraction. Whether VMO activation increases further through additional application of sling-based hip adduction exercise to a knee exercise program is worth exploring.

In summary, the main factor causing patellofemoral pain is an imbalance of VMO and VL muscle, leading to excessive lateral tracking of the patella. VMO strength improvement is the key factor that should be addressed in exercise training. The VMO : VL ratio is also critical because an appropriate ratio can correct patellar alignment and improve VMO strength. The VMO activation is significantly higher during sling-based open and closed kinetic knee extension exercises than during sling-based hip adduction exercises. Sling-based closed kinetic knee extension exercises are significantly more effective in VMO activation than are sling-based open kinetic knee extension exercises. The VL activation induced by the sling-based open and closed kinetic knee extension exercises is significantly higher than that induced by the sling-based hip adduction exercises. The VMO : VL ratio is higher and closer to 1 in the sling-based closed kinetic knee extension exercises than in the sling-based open kinetic knee extension exercises. Although the results indicate that both the sling-based open and closed kinetic knee extension exercises activate the quadriceps, the sling-based open kinetic knee extension exercise is more appropriate and is thus recommended for patients with PFPS.

## 5. Conclusion

The sling-based closed kinetic knee extension exercise produced the highest VMO activation among the three sling-based exercises. Similar to sling-based hip adduction exercise, it also had an appropriate VMO : VL ratio and had beneficial effect on PFPS. Future studies can investigate the clinical effects of combining sling-based closed kinetic knee extension and hip adduction exercises for training patients with PFPS. Moreover, additional studies are required to explore long-term therapeutic effects of the sling-based exercises in patients with PFPS.

## Figures and Tables

**Table 1:** Electromyography analysis of MVC for the sling-based open and closed kinetic knee extension and hip adduction exercises.

	Sling-based open kinetic knee extension exercise			Sling-based closed kinetic knee extension exercise			Sling-based hip adduction exercise		
	VMO (%)	VL (%)	VMO : VL	VMO (%)	VL (%)	VMO : VL	VMO (%)	VL (%)	VMO : VL
Male (*n* = 30)	0.61 ± 0.15	0.76 ± 0.13^‡^	0.81 ± 0.34	0.69 ± 0.19^†^	0.71 ± 0.16^†^	1.01 ± 0.32^*∗*^	0.54 ± 0.13	0.57 ± 0.15	1.01 ± 0.36
Female (*n* = 30)	0.60 ± 0.24	0.76 ± 0.12^‡^	0.78 ± 0.30^‡^	0.72 ± 0.19^*∗*†^	0.74 ± 0.12^†^	0.98 ± 0.25^*∗*^	0.53 ± 0.14	0.55 ± 0.14	1.02 ± 0.61
Total (*n* = 60)	0.60 ± 0.20^‡^	0.76 ± 0.12^‡^	0.80 ± 0.31^‡^	0.71 ± 0.20^*∗*†^	0.72 ± 0.13^†^	1.00 ± 0.28^*∗*^	0.54 ± 0.13	0.56 ± 0.14	1.02 ± 0.35

VMO = vastus medialis oblique; VL = vastus lateralis. No significant differences (*P *> 0.05) were observed in the VMO and VL activities between male and female participants for the 3 exercises.

MVC = (MVC for sling-based exercise/average of maximal isometric MVC test for knee extension) *∗* 100.

^*∗*^Significantly greater muscle activation (*P* < 0.05) compared with the sling-based open and closed kinetic knee extension exercises.

^†^Significantly greater muscle activation (*P* < 0.05) compared with the sling-based closed kinetic knee extension and hip adduction exercises.

^‡^Significantly greater muscle activation (*P* < 0.05) compared with the sling-based open kinetic knee extension and hip adduction exercises.
